# Recent Developments on the Effects of Micro- and Nano-Limestone on the Hydration Process, Products, and Kinetics of Cement

**DOI:** 10.3390/ma17092133

**Published:** 2024-05-01

**Authors:** Xin Li, Mingli Cao

**Affiliations:** School of Civil Engineering, Dalian University of Technology, Dalian 116024, China; lixin_@mail.dlut.edu.cn

**Keywords:** cement hydration, calcium whisker, limestone powder, nano-calcium carbonate

## Abstract

Limestone is commonly used in cement concrete due to its unique nature and type. It has physical effects (nucleation effect and dilution effect) and chemical effects on the hydration process of cement. This paper reviews the effects of three representative limestone materials on the hydration process, hydration products, and hydration kinetics. In the hydration process, the reaction was delayed by limestone powder with a particle size larger than 20 μm and calcium carbonate whiskers due to their dilutive effect. On the other hand, limestone powder with a particle size smaller than 20 m and calcium carbonate nanoparticles facilitated the reaction through nucleation and chemical effects. Limestone has a similar effect on hydration products, promoting the production of C-S-H through nucleation. The mechanism of action for this nucleation effect depends on the differences in crystalline form and particle size of the three types of micro- and nano-calcium. Chemical effects impact the amount of AFt produced, with the generation of new products being the main reaction influenced by the limestone admixture.

## 1. Introduction

Concrete is the most widely used building material globally due to the easy availability of raw materials and low cost. However, the production of cement emits significant amounts of CO_2_ and consumes large amounts of energy, which is harmful to the environment [1,2]. Therefore, researchers aim to decrease carbon emissions and energy consumption in cement production by reducing the amount of cement used in concrete [3,4]. To reduce the amount of cement used, supplementary cementitious materials (SCMs) are commonly employed as an alternative to cement, with fly ash being the most common admixture [5,6,7], silica fume [8], slag [9,10,11], rock dust [12,13], limestone [14,15,16,17], and et al. These SCMs can also enhance various properties of concrete [18]. Limestone is a calcium carbonate sedimentary rock with calcite as its principal component. Calcium carbonate is a compound that includes chalcopyrite, aragonite, and calcite [19]. Among the various forms of calcium carbonate (CaCO_3_), calcite is the most stable at room temperature and pressure. Its crystal system is tripartite, its space group is *R*$\bar{3}$*c*, and its most stable cleavage surface is (10$\bar{1}$4) [20,21]. This cleavage surface is particularly prevalent in geological environments and can be exploited in the production of cement clinkers [22]. Aragonite is a high-pressure phase of calcium carbonate crystals that exhibits an orthorhombic crystal system with a space group of *Pmcn* and a most stable cleavage surface of (001) [21]. It is usually widely distributed as a component of the shells of marine organisms. Depending on the differences in their size [23,24], crystalline shape [25,26], and morphology [27,28], they can play different roles in concrete, such as aggregates [29], fillers [30], and microfibers [31]. Numerous studies have shown that the addition of limestone improves various properties of concrete [14,32]. When used as an aggregate, it can effectively improve the compressive and splitting tensile strength of concrete [33,34], and when used as a filler, it can effectively improve the compactness of the matrix and enhance the performance of concrete [35], including compressive strength and durability [10,36]. Calcium carbonate whiskers (CWs), like other microfibers, can prevent the expansion of microscopic cracks [37,38]. Limestone particles smaller than 1 mm can form a ternary system with other materials that have volcanic ash activity (such as fly ash), which contributes to the hydration reaction of the cement [39]. This, in turn, improves the mechanical properties and microstructure of the concrete [40].

Numerous studies have been conducted to explain the mechanisms by which limestone affects cement hydration. The possible mechanisms that are widely accepted include dilution, nucleation, and chemical action [41]. Dilutive effects are caused by the addition of admixtures, which increase the interparticle distance and dilute the concentration of cement in a certain area. This results in a slower rate of hydration in that area [42]. The nucleation effect refers to the fact that the limestone surface is more likely to provide nucleation sites, attract free Ca^2+^ for nucleation and growth, and facilitate the production of hydration products [20,43,44]. Chemical effects mean that calcium carbonate can react with C_3_A to form monocarbonaluminate and monocarbonaluminate (Hc/Mc) and participate in the hydration reaction [17]. The three types of action described above work together in the limestone–cement system to determine the role of calcium carbonate in the hydration process.

Numerous studies have investigated the impact of calcium carbonate on the hydration process and products. This paper reviews the role and factors influencing the hydration reaction and products of cement by limestone with different morphologies, particle sizes, and crystal types. Additionally, the literature summary presents some expectations for future research. There are still many deficiencies in the studies on the role of micro- and nano-calcium in the hydration of cement. Few studies have investigated the influence of CWs on the phase composition and morphology of the products during the hydration process. There are no systematic studies on the quantitative characterization of the influencing factors of micro- and nano-limestone in the hydration process.

As micro- and nano-calcium are micro-active dopants, the additional nucleation area provided by them is not quantitatively characterized in relation to the enhancement of nucleation rate, which also leads to the lack of accurate quantitative representation of the role of micro- and nano-limestone in the hydration kinetics. The role of calcium carbonate whiskers in hydration kinetics and product morphology has not been specifically analyzed and concluded, and can be studied in more depth in future research.

## 2. Influence Mechanism

Limestone affects the hydration process of cement through dilution, nucleation, and chemical effects. These effects are often interdependent in hydration reactions, and the factors influencing them are complex. This section discusses the modes of action and influencing factors of these effects.

### 2.1. Dilution Effect

**Figure 1:**
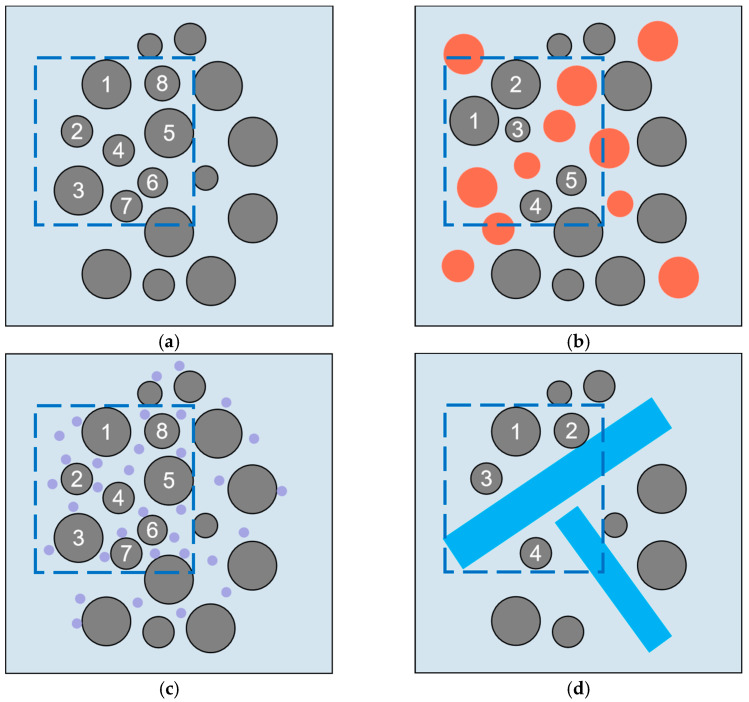
Schematic representation of the dilutive effects for the different blended systems; numbers 1–8 represent the number of cement clinker particles in a fixed area. Orange represents limestone, purple represents calcium carbonate nanoparticles, and blue represents calcium carbonate whiskers. (**a**) Cement; (**b**) Cement–limestone; (**c**) Cement–nano-limestone; (**d**) Cement–carbonate calcium whisker.

The dilutive effect is widespread in mineral admixtures [42,45]. The addition of mineral admixtures typically results in a reduction in cement content, which dilutes the silicate cement content in certain areas [30,42]. This increases the space available for the formation of hydration products, leading to a decrease in the supersaturation of the pore solution required for C-S-H generation [46]. As a result, the cement is more readily hydrated under the influence of the dilutive effect [30]. However, the dilutive effect results in a less compact cement matrix due to the need for higher pore solution supersaturation during hydration in smaller-diameter pores [30,47]. The dilutive effect is solely physical and is influenced primarily by particle size and dosage [32]. Increasing the dosage and particle size enhances the dilutive effect [32,48], as shown in Figure 1. When the average particle size of limestone is larger than that of the cement clinker, the hydration rate is drastically reduced, in which case the dilutive effect does not promote the early hydration reaction [32].

### 2.2. Nucleation Effect

The impact of nucleation on the hydration reaction is typically facilitated in two ways. Firstly, calcite (10$\bar{1}$4) has a surface atomic arrangement similar to that of C-S-H, which enables it to adsorb Ca^2+^ and allow C-S-H to nucleate and grow on its surface [49,50]. Secondly, limestone promotes nucleation by dissolving Ca^2+^ so that C-S-H reaches the required degree of supersaturation for nucleation [32,51]. The addition of calcite enhances the dissolution of alite, which, in turn, promotes the nucleation of C-S-H [32,41]. The two approaches described above work together in the hydration reaction. Calcite promotes C-S-H nucleation, as demonstrated by the calculation of its saturation index. The saturation index of C_3_S is lower than 0.04, while the saturation index of calcite is ≥0.25. This suggests that C-S-H tends to nucleate on the surface of C_3_S only when the SI is lower than 0.04 [50,52]. When the water–cement ratio is between 0.35 and 0.5, the pH range of the pore solution makes C-S-H more likely to undergo heterogeneous nucleation [50,52,53] rather than secondary nucleation on C-S-H or C_3_S surfaces [50]. Therefore, during the experimental process, C-S-H shows a preference for heterogeneous nucleation on the calcite surface. When C-S-H nucleates heterogeneously on the calcite surface, the required degree of supersaturation for C-S-H nucleation is reduced, shortening the induction period [51,54]. Additionally, the presence of limestone provides a larger heterogeneous nucleation surface for the hydrated products, facilitating their nucleation and growth [51].

**Figure 2:**
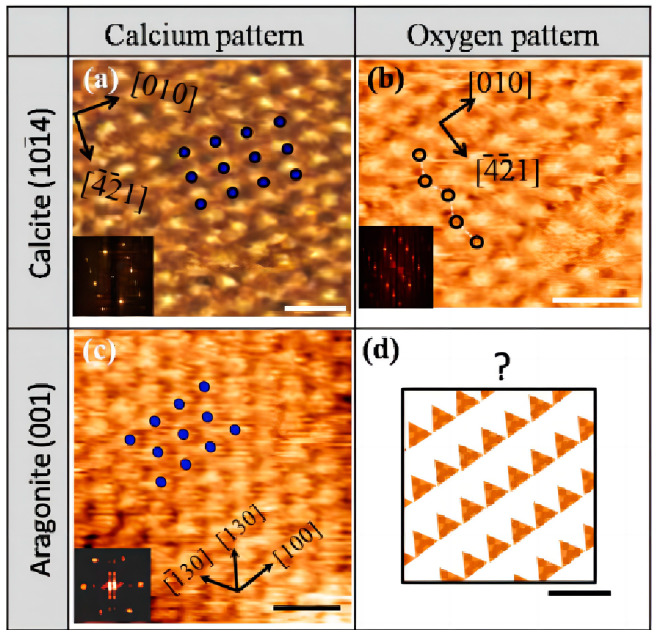
Comparison of atomically resolved and Fourier-transformed images of calcite cleavage (10$\bar{1}$4) and aragonite (001) faces. On the calcite cleavage plane, (**a**) there is a latticed calcium layer and (**b**) a zigzag oxygen pattern, and (**c**) the calcium layer was only observed on the (001) face of aragonite [21], (**d**) oxygen pattern iobserved on the (10$\bar{1}$4) face of aragonite.

**Figure 3:**
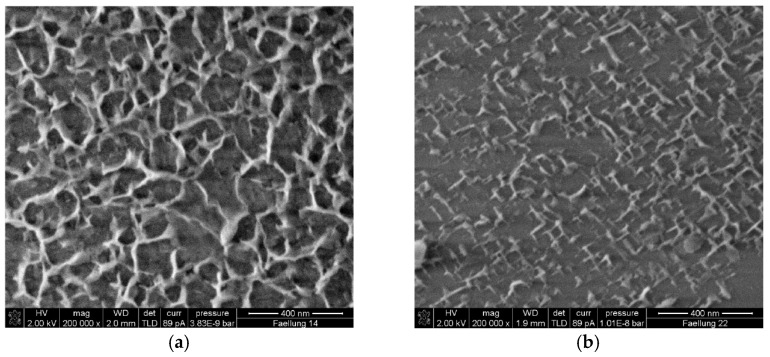
Surface of the calcite particle after the C-S-H growth experiment [49]. (**a**) 0.5 g of calcite; (**b**) 10 g of calcite.

The nucleation effect mechanism suggests that the crystalline form of limestone is the primary factor affecting the nucleation effect. The calcium ions of calcite are in the same surface area as the oxygen ions atoms, so that the arrangement of both types of atoms can be observed simultaneously in the (10$\bar{1}$4) solvation surface, as shown in Figure 2a,b. Calcite has a similar atomic arrangement to that of C-S-H and, therefore, has a more pronounced nucleation [55]. On the other hand, the calcium ions of aragonite are not in the same plane as the oxygen ions, so only the calcium ion arrangement can be observed on the (001) solvation surface and the oxygen ion arrangement cannot be observed, and, therefore, aragonite does not have a specific facilitating effect on C-S-H nucleation [21]. The pseudo-hexagonal pattern in Figure 2d is inconsistent with all the patterns in calcite, which also leads to the very different crystal properties of aragonite and calcite. However, mineral admixtures, such as aragonite, can still facilitate C-S-H nucleation by providing a larger area for hydration product nucleation [56]. In addition to the differences in crystal structure, the amount of limestone doping also affects the nucleation effect. As the amount of limestone doping increases, the nucleation density of C-S-H increases while the size of C-S-H decreases, as shown in Figure 3. Additionally, the amount of C-S-H generated also increases with the increase in doping [49,54].

### 2.3. Chemical Effect

In systems containing limestone, which is slightly soluble in water [57], excessive tricalcium aluminate (C_3_A) can react with it to form hemicarbonaluminate (Hc) and monocarbonaluminate (Mc) [17]. The specific reactions are illustrated in Equations (1)–(3) [58].

C_3_A + CC + 11H → C_4_ACH_11_ (1)

C_3_A + 0.5CC + 0.5CH + 11.5H → C_4_ACH_0.512_ (2)

C_3_A + CaCO_3_ → Mc/Hc (mono/hemicarboaluminate) (3)

**Figure 4:**
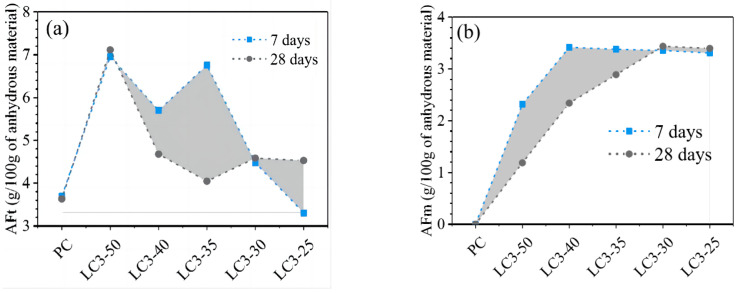
Quantified AFt (**a**) and AFm (**b**) phases of the investigated mixtures based on XRD Rietveld refinement, the gray area represents the amount of change in AFt and AFm from 7 to 28 days [30].

Equation (1) shows that the generation of Hc can consume calcium hydroxide and promote the hydration reaction by reducing the amount of hydration products. Additionally, the presence of limestone allows excessive C_3_A to participate in the reaction to produce Hc and Mc, which prevents the conversion of ettringite (Aft) to AFm and stabilizes Aft [32]. The limited improvement of limestone’s reaction to C_3_A during the later stages of the hydration process [32] is primarily due to the fact that the majority of the C_3_A in pure cement undergoes hydration. The chemical reaction is affected by two main factors: the amount of limestone and C_3_A in the system and the reaction kinetics [39]. The chemical reactions are minimally affected by the properties of the limestone itself, and differences in crystal type and particle size do not have a major effect on the chemical effects [26,32,55]. The aluminum phase content in the system is typically increased using mineral admixtures that are rich in alumina and blended with limestone. This promotes chemical effects [58,59]. The inclusion of the aluminum phase from the mineral admixtures resulted in a significant increase in the production of AFt. Additionally, the production of Mc also increased [30], as illustrated in Figure 4.

### 2.4. Discussion

The impact of the three types of limestone on hydration products was comparable. Nucleation effects promoted the production of C-S-H, while chemical effects influenced the amount of AFt and the production of new products. Although limestone powder and nano-calcium carbonate have similar effects on hydration products, the difference in particle size between the two leads to a difference in the mechanism of influence. The particle size of limestone powder is similar to that of C_3_S. As a result, C-S-H attaches to its surface and grows vertically. This promotes the dissolution of C_3_S and the nucleation of C-S-H. In contrast, nano-calcium carbonates are much smaller than C_3_S particles. Therefore, they can contribute to the secondary nucleation of C-S-H by adhering to the surface of C_3_S and C-S-H, which also results in C-S-H usually having a higher density in the systems containing nano-calcium carbonate.

## 3. Influence of Limestone on Hydration Reactions

Limestone with a particle size below 1 mm generally plays a diluting, nucleating, and chemical role in the cement hydration process [59,60]. Dilution usually slows down the hydration rate and delays the exothermic hydration starting [26,32]. The nucleation effect usually results in an increase in the hydration rate with an increase in the peak exothermic value of hydration [16,61,62].

### 3.1. Limestone Powder (LP)

Limestone powder (LP) typically has a particle size between 10 and 100 μm, which is smaller than the average particle size of a cement clinker [16]. LP can, therefore, affect the hydration reaction through physical and chemical effects. Table 1 presents a review of the impact of varying sizes of limestone powder (LP) on the cumulative heat release, peak hydration heat release, and peak occurrence time.

Table 1 shows that dosage and particle size are the main factors influencing the exothermic hydration in LP. For LP with a particle size below 20 μm, appropriate doping can increase the total amount of exothermic hydration, increase the peak rate of exothermic hydration, and advance the time of peak exothermic hydration [7,45,64]. Aqel et al. [64] demonstrated that the heat of hydration increased with the addition of LP, and this increase was inversely proportional to the particle size. The hydration reaction was promoted by LP, resulting in a shorter induction time, mainly due to the nucleating effect, leading to the second exothermic peak advancing [65,66]. However, for LP with a particle size larger than 20 μm, the addition will significantly reduce the hydration exotherm [16,26,32], and variations in the above results are mainly due to LP particle size selection. Briki et al. [32] conducted a study on the effect of LPs with different finenesses on early hydration. Results showed that the packing effect of LPs with a particle size of 2 μm compensates for the dilution effect, which is attributed to the fact that finer LPs increase the undersaturation of alite, which results in a faster dissolution rate of alite and promotes the nucleation effect of C-S-H on the surface of LPs [41,52], leading to higher exothermic hydration of alite.

**Figure 5:**
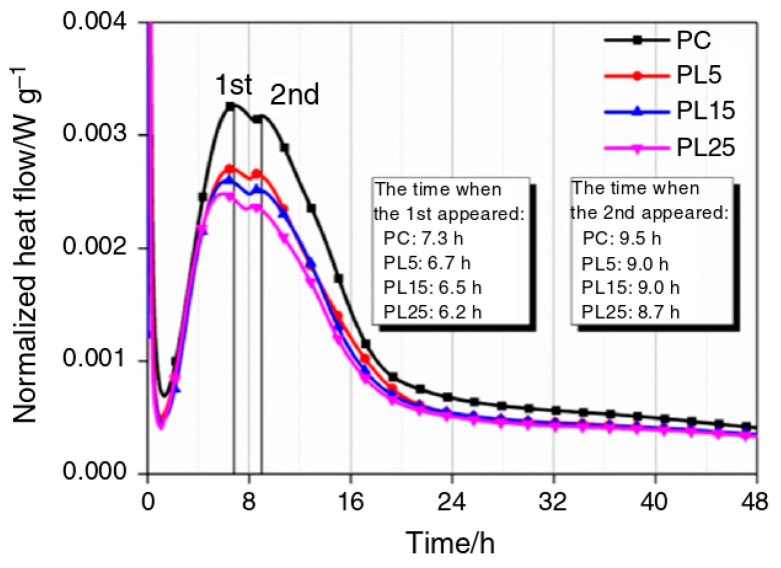
Hydration heat flow curve of the LP Portland cement (PC) binary system [16].

After the dosage of LP exceeded 20 wt%, the peak rate of the exothermic rate of cement hydration decreased, and the time of peak appearance was delayed [16,25,66], as shown in Figure 5. This reduction in the exothermic rate of hydration was mainly due to the diluting effects of LP [25,39]. Although LP provides more nucleation sites for C-S-H with increasing doping, the addition of LP also increases the effective water–cement ratio [25]. This dilutive effect outweighs the nucleation effect [32]. However, increasing the dosage also promotes chemical effects and the production of Hc and Mc, which favors the formation of hydration products [16].

### 3.2. Calcium Carbonate Whiskers (CWs)

Calcium carbonate whiskers are typically fibrous, with diameters ranging from 0.5 to 2 μm and lengths from 20 to 30 μm [67]. The crystalline form of these whiskers is usually aragonite, which can undergo a transformation to calcite at 450 °C [68,69]. Calcite whiskers were obtained by Li et al. through high-temperature treatment of the CW. The temperature was raised to 450 °C, kept for 2 h, and then cooled naturally [26]. For the reaction peak of alite, the calcite CW accelerated the appearance time of its peak and increased the intensity of the exothermic peak of the C_3_S reaction compared to the aragonite CW. However, for the second exothermic peak, the peak intensities and appearance times of the two were similar.

**Figure 6:**
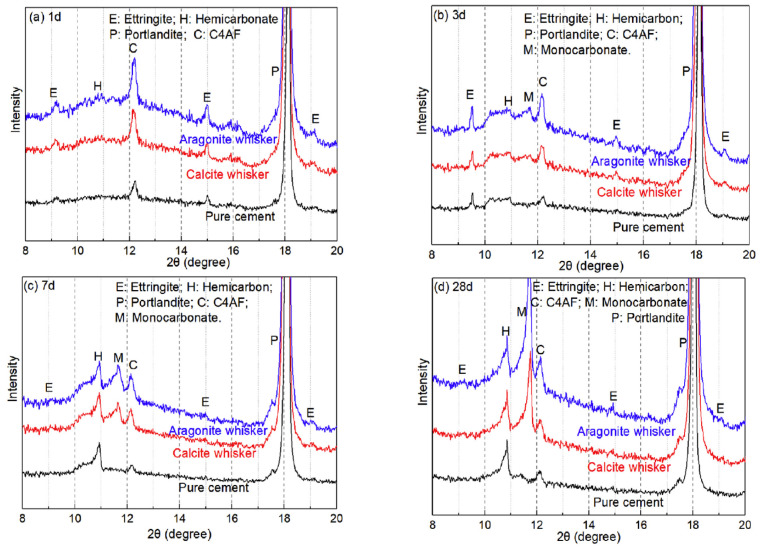
XRD patterns of aragonite and calcite whisker-reinforced cement paste [26]. (**a**) 1 d of hydration; (**b**) 3 d of hydration; (**c**) 7 d of hydration; (**d**) 28 d of hydration.

It has been demonstrated that, similar to larger LPs, the addition of CWs mainly has a dilutive effect and reduces the exothermic heat of hydration. This suggests that the length dimension of the CWs is the main factor controlling the variation of the heat of hydration. Li et al. [26] used XRD to determine the chemical products, Hc and Mc, as shown in Figure 6. The peak intensities of both were found to be essentially the same, which is consistent with the results of the hydration exotherm. Therefore, it can be concluded that crystallographic differences primarily affect the hydration reaction through physical effects, which influence the nucleation of C-S-H on the CW surface. This suggests that differences in crystallographic shape have minimal impact on the chemical effects [26,30].

### 3.3. Nano-Calcium Carbonate (NC)

The size of calcium carbonate nanoparticles typically ranges from 10 to 105 nm, with irregular shapes and an average size of approximately 50 nm. The particles are typically cubic or connected in agglomerated chains [24,70], and XRD test results indicate that the primary constituent is calcite [24]. Currently, some researchers have chosen to pass gaseous CO_2_ during the mixing process to produce calcium carbonate nanoparticles. These are known as in situ grown calcium carbonate nanoparticles (in situ NC), and in addition to solid-state calcium carbonate nanoparticles [71], they increase the density of hydration products [72]. However, the addition of CO_2_ only slightly enhances cement hydration exotherm, which suggests that in situ NC does not significantly affect hydration exotherm [73]. This may be due to the fact that in situ NC generation occurs simultaneously with the hydration reaction.

The addition of NC can dramatically increase the exothermic rate of hydration while advancing the appearance of the alite reaction peak and shortening the induction period [74]. NC has a high surface energy, which can promote ion migration by adsorbing Ca^2+^ released from C_3_S dissolution and thus shorten the induction period [24,74,75]. The incorporation of NC results in a reduction of approximately four hours in the dormant period [75]. Additionally, NC can act as a nucleation site for C-S-H, promoting its growth and playing a nucleation effect [74]. However, the high surface energy of NC makes it prone to agglomeration in the cement matrix, which negatively affects the properties of cementitious materials [9,76]. Differences in the mode of dispersion also led to changes in the early hydration kinetics, with ultrasonically dispersed NC facilitating the hydration reaction [77]. When NC is more uniformly dispersed, it can promote early hydration, increase the hydration rate, and increase the degree of hydration (Doh) [78]. Currently, the recommended dosage of NC in cementitious materials ranges from 1 wt% to 4 wt% [9,79]. Excessive NC dosage can result in agglomeration, preventing proper dispersion and leading to lower exothermic values [61,75]. It has been demonstrated that ultrasonication-dispersed NC can enhance hydration by providing additional nucleation sites [80]. Furthermore, NC can also have a chemical effect by dissolving CO_3_^2−^ to react with C_3_A [24]. Wu et al. [81] also found that chemical effects can advance the second exothermic peak and increase the exothermic values of hydration.

### 3.4. Comparison of the Effect of Different Calcium Carbonates on Hydration Reactions

Table 2 presents a detailed comparison of the primary functions of various forms and grain sizes of limestone in the process of hydration. The presence of the nucleation effect in limestone is widespread when the particle size is below 100 μm. The surface composition of limestone is more similar to that of C-S-H gels [20,62] compared to quartz, making it more susceptible to the adsorption of hydration products for nucleation [32]. Additionally, the inclusion of limestone promotes the easier dissolution of C_3_S, which, in turn, enhances hydration reactions [32]. The dilution effect typically occurs in LP and CWs when using high dosages and large particle sizes. According to Ahmed et al. [30], the promotion of the hydration reaction through filling and nucleation effects is offset by the dilution effect when replacing more than 30wt% of the cement proportion. When the average particle size of LP is larger than that of the cement clinker, its dilutive effect becomes more apparent in the hydration reaction, resulting in a decrease in the rate of the reaction [32]. The atomic arrangement of aragonite and calcite has a significant impact on the hydration reaction, with the crystalline form of limestone also contributing to the hydration reaction, which is mainly due to differences in the atomic arrangement of the two decisions. Calcite has a Ca and O atom arrangement more similar to that of the C-S-H gel surface, which can attract free Ca^2+^ to nucleate and grow and promote the generation of C-S-H [20,43,44]. The effect of aragonite on the hydration reaction proceeds mainly through the dissolution of CO_3_^2−^ for chemical reactions [26,55].

## 4. Effect of Calcium Carbonate on Hydration Products

The effects of calcium carbonate on the hydration reaction alter the type and morphology of the hydration products. The morphology of the hydration products has been influenced by physical effects. Nucleation effects have promoted the generation of hydration products [32], while dilutive effects have negatively impacted the compactness of the cement matrix [30]. Chemical effects generate Hc and Mc, which also grow in the cement matrix, altering the microscopic morphology of the hydration products [32,84].

### 4.1. Limestone Powder (LP)

**Figure 7:**
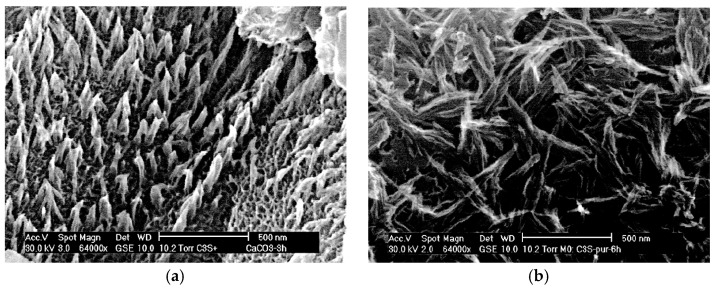
The microstructure of C-S-H during the hydration of C_3_S and calcite powder [51]. (**a**) Growth of C-S-H on calcite; (**b**) Growth of C-S-H on C_3_S.

The addition of LP may impact the hydration products in terms of physical and chemical effects. When the nucleation effect dominates, the production of C-S-H increases. The addition of LP refines the hydration products [84,85], resulting in shorter and coarser needles and rods of C-S-H [15]. In contrast, if the dilution effect dominates, the compactness of the cement matrix decreases, leading to a significant reduction in C-S-H production [30]. In contrast to the C_3_S surface, C-S-H on the calcite surface exhibits oriented growth, as depicted in Figure 7. This growth is primarily attributed to the nucleation effect of calcite [51]. Furthermore, the addition of LP reduces the production of CH, which gradually decreases with increasing LP doping [35,86].

The chemical effects of LP result in the production of Hc and Mc, which consume excess C_3_A [32]. However, it should be noted that Hc is not always stable and can decrease as the reaction age increases [16]. Additionally, the content of Hc can be stable when the content of blended calcite-type LP is lower than 2–3 wt% [84]. Mc and Hc are generated in the interfacial zone between the LP and the cement matrix, forming firmly consecutive crystalline aggregates [84]. Furthermore, the chemical effects stabilize the presence of AFt and prevent its conversion to AFm [30,87,88]. This is advantageous for the properties of cementitious materials, as AFt has a higher strength and larger solid phase volume. To stabilize AFt and promote chemical effects, researchers often elect to introduce alumina-rich admixtures, such as metakaolin, fly ash, and calcined clay, to partially replace cement by blending with LP [63,65,88]. In such ternary systems, the dilution effect is not obvious and, in addition, since the generation of Hc and Mc requires the consumption of CH, it will promote hydration reactions, thus increasing the hydration of the cement clinker [30] and making the cement matrix denser.

The incorporation of LP into hydration products resulted in a decline in compressive strength, which became more pronounced with an increase in LP dosage. The 28d compressive strength of the PC-LP system was observed to be inferior to that of the control when the LP dosage exceeded 20 wt% [14]. This phenomenon can be attributed to the dilution effect of a high dosage of LP, which plays a dominant role in the dilution process [30,64]. When SCMs with volcanic ash activity are co-mingled with LP to participate in the hydration reaction, a small increase in the early compressive strength of this ternary system is observed due to the synergistic effect of the nucleation surface provided by LP [7,11]. This happens while the SCMs increase the activity of LP and enhance the hydration process [10].

### 4.2. Calcium Carbonate Whiskers (CWs)

**Figure 8:**
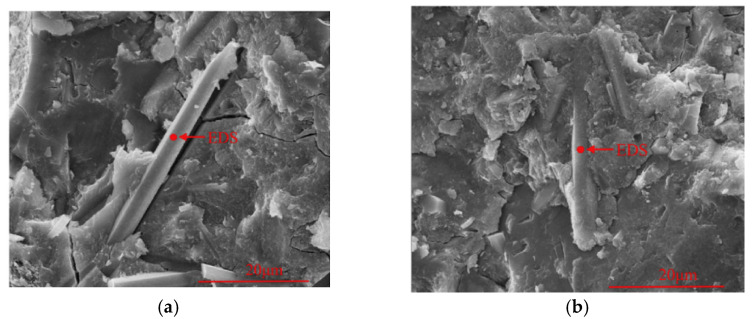
Microstructure of CW-reinforced hardened cement paste [26]. (**a**) 10% aragonite type CW; (**b**) 10% calcite type CW.

CW bridges and deflects microcracks in the cement matrix, resulting in a denser matrix [27,89]. Furthermore, the inclusion of the CW decreases the orientation index of CH, indicating that it restricts the area available for CH crystal growth and improves the density of the cement paste. When comparing the calcite CW with the aragonite CW, it can be observed that the calcite CW has a rougher surface with more hydration products than the surface of the aragonite CW [26], as shown in Figure 8. The CW also has chemical effects and can react with C_3_A to produce Hc and Mc during hydration [26,55]. Additionally, aragonite, which is more soluble than calcite [69], can dissolve more CO_3_^2−^ and participate in the chemical reactions. The CW also has synergistic effects with alumina-rich materials. It provides a calcium source for the volcanic ash reaction of fly ash, while fly ash also provides aluminates for the chemical effects of the CW [82].

The addition of the CW has a small effect on C-S-H generation, and due to its larger volume, it mainly acts as a diluent and reduces the concentration of the cement clinker, as the compressive strength decreases with increasing CW dosage [26,37,82]. The calcite whiskers obtained after the treatment had the most obvious effect on compressive strength improvement [26]. When the CW addition was 40 vol%, the strength was reduced by about 18.65% [31]. A total of 10% of the CW can increase the 28d compressive strength of mortar by 13%, effectively improving the physical and mechanical properties of mortar [90]. The CW is more often used with the rest of the fibers to prepare multi-scale hybrid fiber-reinforced concrete (MHFRCC) [91]. The CW is more commonly used with other fibers in the production of multi-fiber hybrid-reinforced concrete (MHFRCC) [38]. The commonly used fibers include polyvinyl alcohol (PVA) fibers [91,92], steel fibers (SFs), and hybrid fibers (HFs) [93], and the combination of the CW and PVA fibers with steel fibers can effectively improve the mechanical properties of MHFRCC under static loading and also increase the compressive strength of the mortar [91,94]. For hybrid fibers, the CW has good synergy with hybrid fibers of different lengths, and 10% of the CW can increase the compressive strength by about 7% [93]. However, by adding 1% of the CW, 0.45% HF, and 0.36% SF, the compressive strength can be increased by 43% compared to PC [95].

### 4.3. Nano-Calcium Carbonate (NC)

The effect of NC on hydration products is similar to that of LP, which can stabilize the presence of AFt by chemically generating Hc and Mc and consuming CH to promote the hydration reaction to proceed [39]. At lower levels of NC agglomeration, NC promotes early hydration reactions and the generation of C-S-H [78]. The addition of NC also affects the growth of CH by decreasing the growth of CH{001} facets and accelerating the growth of CH{101} facets, leading to a decrease in hexagonal plates and an increase in the amount of prismatic CH [24]. On the other hand, the addition of NC increased the CO_3_^2−^ content in the cement matrix, and the dissolution of CO_3_^2−^ could replace SO_4_^2−^ in AFt, forming a crystal structure similar to that of AFt [24].

NC is connected to the hardened cement paste with a smaller interfacial transition zone [9,96]. The nucleation of NC on the surface of C-S-H gels promotes the secondary generation of C-S-H gels [97]. This also allows the twice-generated C-S-H gels to be cross-coated with the hydration products of NC, resulting in the generation of ultra-high-density C-S-H gels [24]. This reduces the percentage of low-density C-S-H gels and improves the bulk density of the gels [98]. The effect is more pronounced in the early stages of cement hydration [24,99].

The addition of NC improved the compressive strength as it promoted the formation of ultra-high-density C-S-H gels [24]. With increasing dosage, the compressive strength showed a tendency to increase and then decrease, and when the dosage was greater than 4 wt%, the compressive strength of the NC cement system was lower than that of PC [76,80,100]. When the dosage of NC was 3.2 wt%, its effect on compressive strength was most obvious [96,101], and it improved the strength growth rate by 1-3d [81,100]. Since the effect of NC on strength was mainly expressed by the increase in C-S-H density, it mainly changed the compressive strength in the pre-hydration period, and the compressive strength was basically unchanged after 28d [81].

**Figure 9:**
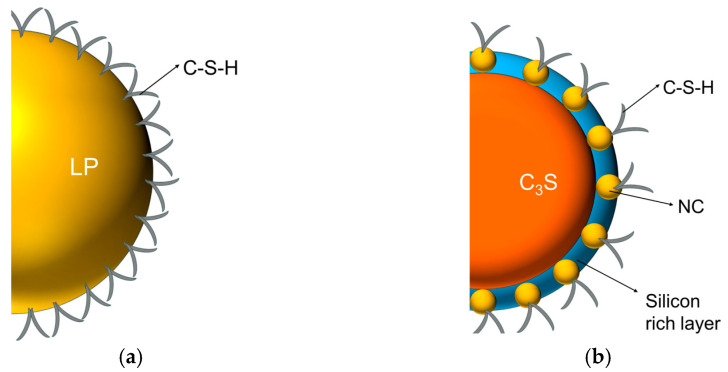
Schematic representation of the nucleation of C-S-H in limestone powder and nano-calcium carbonate. (**a**) Limestone powder (LP); (**b**) Nano carbonate calcium (NC).

By comparing NC and LP, it is possible to observe the difference in the effect of micron-sized limestone and nanosized limestone on hydration products, as illustrated in Figure 9. For LP, the particle size is comparable to that of C_3_S. Therefore, C-S-H will adhere to its surface and grow vertically until the C-S-H size reaches a critical length of about 400 nm [51]. Because the surface of calcite is more prone to act as a nucleation site, the density of C-S-H growth on the surface of C_3_S is lower, and the surface of C_3_S is not fully covered by hydration products [49,51]. This makes it easier to dissolve. Compared to systems without limestone, the rate of hydration reaction is faster. The effect of NC on nucleation is mainly reflected in its ability to destroy the silicon-rich layer on the surface of C_3_S while reducing the ionic concentration around the silicon-rich layer, thus shortening the induction period and promoting the hydration reaction, and at the same time, it can also adhere to the surface of C-S-H and promote the nucleation of C-S-H [24]. The addition of NC typically results in the growth of more C-S-H on its surface, with nucleation occurring on the C_3_S surface. This leads to the formation of denser C-S-H gels.

### 4.4. Effect of Limestone on Durability

The primary component of limestone is calcium carbonate (CaCO_3_), which results in limestone primarily influencing the sulfate resistance of cementitious materials [102]. Sulfate reacts with the C-S-H gel produced by cement hydration to form products, such as calomel and gypsum, in a humid environment in the presence of CO_3_^2−^, leading to expansion and cracking of cementitious materials [102,103]. Limestone provides carbonate ions for this process, and thus the addition of LP generally has a detrimental effect on the resistance of cementitious materials to sulfate attack [104]. When the LP dosage is increased to 15 wt%, it has a noticeable effect on the durability of the cementitious materials [103]. The expansion rate was faster, and the damage occurred earlier than in PC [103,104]. Low temperatures also result in a reduction in the sulfate attack resistance, which is facilitated by a slight increase in CO_2_ at 0–5 °C, leading to an increase in the CO_3_^2−^ content [36,103]. The sulfate attack resistance of the LP-PC system can be effectively enhanced by the incorporation of the remaining SCMs [105]. Following the addition of metakaolin, the concrete samples subjected to sulfate attack, and the overall properties and apparent morphology exhibited significant improvements in comparison to those of PC and LP-PC [106].

In addition, chloride ion permeability is an important index for evaluating the durability of cementitious materials. The increase in LP doping leads to a deterioration of the chloride ion permeability of the cementitious materials [106]. Nevertheless, when chloride ions and sulfate ions are present simultaneously, the presence of chloride ions reduces the extent of sulfate attack. The complication of the co-existence of Cl^−^ and SO_4_^2−^ is mainly found in seawater erosion. Furthermore, chloride ions penetrate deeper into the matrix than sulfate ions [107]. In addition to chloride erosion, Nadelman et al. [108] investigated the impact of limestone on the physical salt erosion induced by the addition of LP at higher water–cement ratios (0.6). This resulted in the refinement of the pore structure and the observation of more expansion cracking due to salt crystallization. In addition to salt attack, the performance of cementitious materials under high-temperature conditions is also an important index for evaluating their suitability for such conditions [39]. The incorporation of NC and CWs can effectively improve the performance of cementitious materials after high temperatures [109,110].

## 5. Numerical Modeling of Hydration

Numerical simulation can be used to explore the hydration process by calculating the hydration reactions, determining the roles of each reactant in the process [111,112,113,114], identifying the factors that control the transformation of each hydration process [115], and understanding the mechanism by which external factors or admixtures influence the hydration process [40,48]. Thermodynamic calculations are the main method used today for the numerical modeling of limestone hydration.

### 5.1. The Thermodynamics of Hydration

Thermodynamic calculations can provide a reliable representation of the phase composition and the chemical composition of a system at a given temperature and pressure [116], characterize the effect of the external environment on the phase composition of the cement and hydration products, and determine the degree of cement hydration at the time of interest [115]. Currently, the main software used includes ① GEMs, and the corresponding databases include Cemdata07 [113,117], Cemdata14, and Cemdata18 [116]. ② PHREEQC version 3 software, as a commonly used geochemical calculation software, can also be used to calculate the thermodynamics of cement hydration [118,119], which mainly uses the PHREEQC database with the HATCHES database [120].

**Figure 10:**
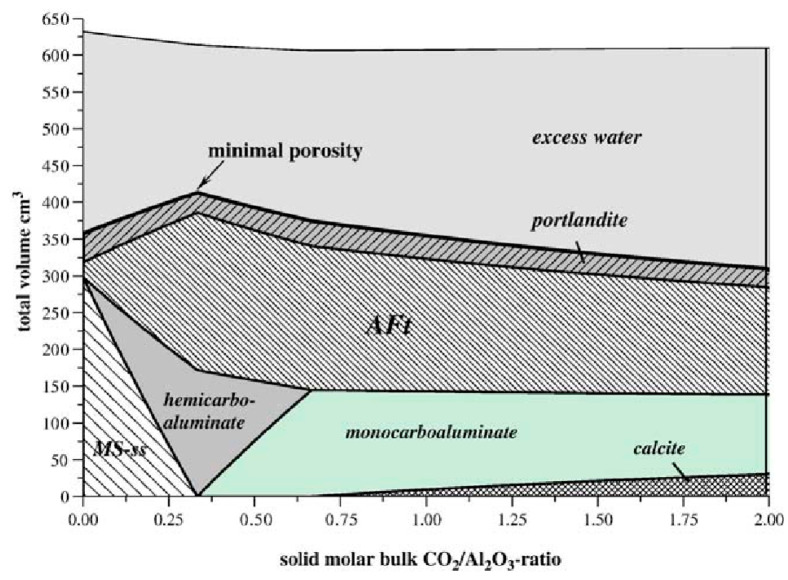
Variation of hydration products with the CO_2_/Al_2_O_3_ ratio under constant total solid conditions [17].

GEM computational simulations support the computational simulation of many hydration processes, including geopolymers, ordinary Portland cement [115], and Portland cement [121]. Matschei [17] carried out thermodynamic calculations on the hydration reaction system involving calcite and determined that calcite is involved in the system, as shown in Figure 10. Initially, the formation conditions of Hc and Mc were derived. Based on this study, some researchers have further investigated the specific generation conditions and existence states of Hc and Mc using GEMs [116,119], which showed that the chemical compositions of Hc and Mc are independent of the CaCO_3_ content [116] and that Hc and Mc can be further converted to hydrogarnets [113].

### 5.2. Hydration Kinetics

**Figure 11:**
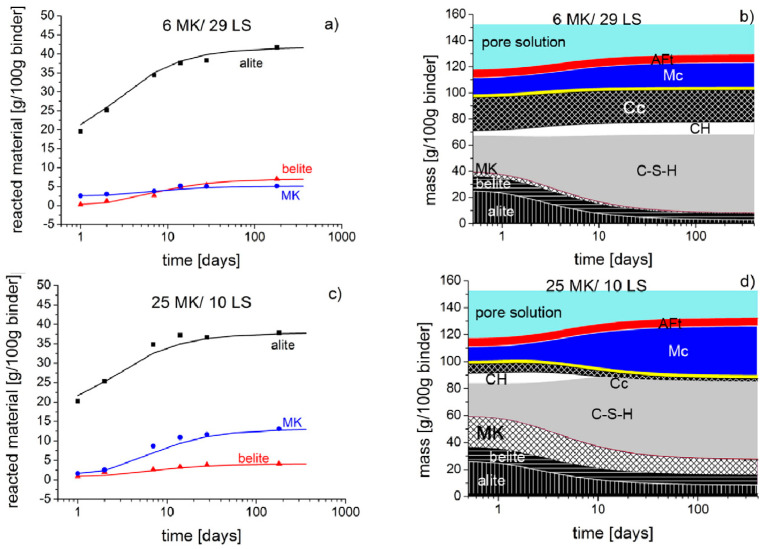
Comparison of the experimental (symbols) and modeled (lines) quantities of reacted alite, belite, and MK for 1 day to 182 days of hydration with (**a**) 6 wt% metakaolin and 29 wt% limestone powder, and (**c**) 25 wt% metakaolin and 10 wt% limestone powder. The composition of hydration products for 1 day to 182 days is shown in (**b**,**d**) [111].

Blending limestone with other SCMs that are rich in aluminum and partially replacing cement can result in significant performance gains due to the chemical effects of limestone [122]. Further research was conducted to investigate its effect on hydration kinetics and its role in ternary systems [30,40]. Kunther et al. [111,123] used ^27^Al MAS NMR and ^29^Si MAS NMR to determine the products at different reaction ages based on GEMS. They optimized the results of the thermodynamic tests and proposed to carry out the reaction kinetics calculations of the components in the ternary system. These calculations can be used to estimate the extent of the reaction, with the equations shown in Equations (4)–(6) [40,111]. Combining kinetic calculations with GEMs effectively improves the accuracy of thermodynamic calculations, and experimental results fit well with simulation results [123]. Figure 11 shows the kinetic calculations for ternary systems, which typically focus on the generation of hydration products at different reaction ages. Alite has the highest degree of hydration and the fastest rate of increase in hydration for the same hydration age when metakaolin (MK) and limestone (LS) masses are constant. Blite has a slower hydration reaction and a low degree of hydration [111]. The degree of hydration and the rate of reaction of MK, on the other hand, increased as the mass of LS increased, mainly because MK would react with LS to promote hydration [88,111]. The results indicate that when there is a sufficient alumina phase in the system, the hydration products are dominated by C-S-H gels. This is because the later reaction between the biotite and limestone consumes part of CH [86].

(4) $$Q_{i}(t)=Q_{i0}+{\bar{k}}_{i}exp\cdot ({-n}_{i}/t)$$

(5) $$Q_{i0}=q_{i}\cdot \alpha \left(t_{1}\right)$$

(6) $${\bar{k}}_{i}=q_{i}\cdot \left[\alpha \left(t_{x}\right)-\alpha \left(t_{1}\right)\right]$$

where *i* is the reactant, *Q_i_(t)* and *Q_i_*_0_ are the reactants *i* at time *t* and at the start of the reaction, respectively, *k_i_* is used to limit the possible dissolved mass during the reaction, *n_i_* is the hydration rate parameter, *q_i_* is the initial dissolved amount, and $\alpha (t_{x})$ is the degree of hydration at the end of the reaction.

## 6. Application Trend of Limestone

Currently, limestone is more commonly blended with clay and cement and used as LC3 instead of cement [124]. The preparation process of LC3 has lower carbon emissions [125], while the mechanical properties of LC3 are more excellent [126,127]. Furthermore, due to the lower content of cement in LC3, less CH is produced by hydration, which makes the durability performance of LC3 concrete better than that of cement concrete [126].

Theodore and colleagues demonstrated that the production cost of LC3 is approximately 10–20% lower than that of silicate cement, with the potential for further reduction if industrial wastes are employed in lieu of clay [128]. Additionally, the exceptional durability of LC3 will result in a reduction in the frequency of building repairs and lower maintenance costs [90]. In addition to LP, the application of the CW as a microfiber can also reduce the cost of multi-scale hybrid fiber concrete [92]. The CW is manufactured at a cost of approximately USD 200 per ton [129], so the CW can partially replace the more expensive PVA fiber with steel fiber, reducing costs [91]. Meanwhile, the inclusion of microfibers can stop the growth of microcracks in concrete in a limited way and improve the flexural properties of concrete [130]. In addition, the production cost of NC is lower compared to other nanomaterials, while NC is compatible with cementitious materials [131]. Therefore, NC is also a common choice when it comes to nano-enhancement [132]. However, nanomaterials need to be dispersed before they can exert a better reinforcing effect [79,97], and the engineering practical application is less at present.

The current application of limestone in engineering practice is primarily for use as an admixture to partially replace cement or as a component of LC3 [133,134]. Furthermore, LC3 is now also used in the construction field in the initial application. The model house in Jhansi, India, employs LC3 as a component of the cementitious material, utilizing 26.6 tons of industrial waste, a process that reduces CO_2_ emissions by 15.5 tons. Additionally, in India, LC3 is utilized in the construction of road pavements [135]. In Latin America, LC3 is employed in a variety of settings, including buildings, offshore test sites, artistic sculptures, and pavements. The LC3 House in Santa Clara, Cuba, is an example of a structure that emits 30% less CO_2_ during production compared to conventional concrete [135].

## 7. Conclusions and Outlook

### 7.1. Conclusions

This review examines the impact of limestone with varying morphologies, crystal types, and grain sizes on hydration reaction, products, and kinetics, as well as the factors that influence them. From this analysis, the following conclusions can be drawn:

(1) The effects of limestone on the hydration reaction can be divided into two categories: promotion through nucleation and chemical effects and delay through dilution. As the dosage and particle size of limestone powder increase, the dilutive effect becomes more pronounced. Calcium carbonate whiskers, due to their large size, primarily have a dilutive effect on the hydration reaction. Nano-calcium carbonate promotes the hydration reaction through nucleation and chemical effects due to its small particle size. The differences between the three types of limestone mentioned are attributed to variations in particle size and crystal type. Particle size affects the dilutive effect, while crystal type has a greater impact on the nucleation effect.
(2) With regard to LP, its addition primarily served to enhance the filling and nucleation effects, thereby facilitating the generation of C-S-H and improving the compressive strength. In the case of the CW, its principal role was that of a microfiber, which reduced the development of microcracks and improved the mechanical properties of cementitious materials. As NC can play a more significant filling role in smaller pores, it promotes the generation of C-S-H with CH and improves mechanical properties.
(3) Numerical simulations of hydration in multifaceted systems containing limestone typically employ thermodynamics, such as GEMs, to predict and simulate the composition of hydration products at different ages. Empirical formulas are still mostly used for the kinetic calculation of the multivariate system, and the thermodynamic fitting results are more accurate after incorporating the kinetic calculation.
(4) There are three primary mechanisms by which limestone affects cement hydration. The first is the dilutive effect, which occurs when limestone is added to the mixture, reducing the cement content in a particular region, increasing the space available for the growth of hydration products, and promoting the hydration reaction and product formation. (2) The nucleation effect has two main aspects: ① the attraction of calcite to Ca^2+^ and ② the reduction in the supersaturation degree of the solution required for C-S-H nucleation. Therefore, the promotional effect of aragonite on C-S-H nucleation is much weaker than that of calcite. (3) The chemical effects of the reaction between limestone and excessive C_3_A to produce Hc and Mc and to stabilize the AFt produced by hydration, the main factor influencing the chemical effects was the addition of limestone.

### 7.2. Outlook

The advent of contemporary technological innovations has led to a surge in the availability of micro- and nano-limestone. In situ NC is effectively dispersed, while the process utilizes CO_2_ emitted from cement production, thereby enhancing the sustainability of the concrete preparation process. However, this approach is currently constrained to the production of calcite, and the process itself limits further investigation into the impact of nano-aragonite on the hydration reaction.

The main national standards and regulations for limestone cement set limits on the amount of limestone that can be used. However, these standards do not address the differences between different types of limestone in the application process, and future recommendations should be tailored to the different types of limestone.

## Figures and Tables

**Table 1:** Exothermic changes in hydration.

Reference	W/C	Particle Size	Dosage	Cumulative Heat Release	Peak Heat of Hydration	Appearance Time
[16]	0.5	10–100 μm	5 wt%	Reduced by 6%	-	About 0.6 h ahead of schedule
			15 wt%	Reduced by 18%	-	About 0.8 h ahead of schedule
			25 wt%	Reduced by 17%	-	About 1.1 h ahead of schedule
[45]	0.4	6.5 μm	10, 20, 30, 40, 50 wt%	85% increase at 30 wt%	About 28% improvement at 30 wt%	About 2.5 h ahead of time
		3.5 μm		71% increase at 30 wt%	About 30% improvement at 30 wt%	About 3.5 h ahead of schedule
		9 μm		Approx. 63% improvement at 30 wt%		About 2.5 h ahead of time
		3 μm		-	About 5% improvement at 10% dosing	
		15 μm		-	Improvement of about 1% at 10% dosing	Approximately 1% ahead of schedule
[32]	0.4	2 μm	20 wt%	-	Improvement of about 11%	Approximately 13% ahead of schedule
		130 μm		-	Reduction of about 19%	Delayed by about 16%
[63]	0.4	1–10 μm	25 wt%	Improvement of about 28%	Improvement of about 32%	Approximately 19% ahead of schedule
			50 wt%	Improvement of about 19%	Improvement of about 42%	Approximately 44% ahead of schedule

**Table 2:** Effect of micro- and nano-calcium carbonate on cement hydration.

Reference	Type	Particle Size	Dosage (wt%)	Mechanism of Action
Cao et al. [37]	CW	length 20–30 μm, diameter 0.5–2 μm	5%, 10%, 15%, 20%	nucleation effect
Ming et al. [82]	CW	length 20–30 μm	10%	nucleation effect, chemical effect
Sato et al. [61]	NC	50–120 nm	10%, 20%	nucleation effect, chemical effect
Ouyang et al. [28]	LP	9 μm	30%	nucleation effect, dilutive effects
Aqel et al. [69]	LP	17 μm,12 μm, 3 μm	5%, 10%, 15%	dilutive effects
Berodier [83]	LP	2 μm, 15 μm,	40%	nucleation effect, dilutive effects
Zemei Wu et al. [81]	NC	15–105 nm	1.6%, 3.2%, 4.8%, 6.4%	nucleation effect
Li et al. [26]	aragonite CW	length 20–30 μm, diameter 0.5–2 μm	5%, 10%	nucleation effect, chemical effect, dilutive effects
	calcite CW			

## Data Availability

The data in this article cannot be shared directly for article type reasons.

## References

[1] Ashraf W., Olek J., Jain J. (2017). Microscopic features of non-hydraulic calcium silicate cement paste and mortar. Cem. Concr. Res..

[2] Yang K.-H., Jung Y.-B., Cho M.-S., Tae S.-H. (2015). Effect of supplementary cementitious materials on reduction of CO_2_ emissions from concrete. J. Clean. Prod..

[3] Selim M., Khalifa R., Elshamy E., Zaghlal M. (2024). Structural Efficiency of Fly-Ash Based Concrete Beam-Column Joint Reinforced by Hybrid GFRP and Steel Bars. Case Stud. Constr. Mater..

[4] Kanagaraj B., Kiran T., Gunasekaran J., Nammalvar A., Arulraj P., Gurupatham B.G.A., Roy K. (2022). Performance of Sustainable Insulated Wall Panels with Geopolymer Concrete. Materials.

[5] Moudio A.M.N., Tchakouté H.K., Ngnintedem D., Andreola F., Kamseu E., Nanseu-Njiki C., Leonelli C., Rüscher C. (2021). Influence of the synthetic calcium aluminate hydrate and the mixture of calcium aluminate and silicate hydrates on the compressive strengths and the microstructure of metakaolin-based geopolymer cements. Mater. Chem. Phys..

[6] Miao X., Pang X., Li S., Wei H., Yin J., Kong X. (2023). Mechanical strength and the degradation mechanism of metakaolin based geopolymer mixed with ordinary Portland cement and cured at high temperature and high relative humidity. Chin. J. Chem. Eng..

[7] Vance K., Aguayo M., Oey T., Sant G., Neithalath N. (2013). Hydration and strength development in ternary portland cement blends containing limestone and fly ash or metakaolin. Cem. Concr. Compos..

[8] Muller A.C.A., Scrivener K.L., Skibsted J., Gajewicz A.M., McDonald P.J. (2015). Influence of silica fume on the microstructure of cement pastes: New insights from 1H NMR relaxometry. Cem. Concr. Res..

[9] Hosan A., Shaikh F.U.A., Sarker P., Aslani F. (2020). Nano- and micro-scale characterisation of interfacial transition zone (ITZ) of high volume slag and slag-fly ash blended concretes containing nano SiO_2_ and nano CaCO_3_. Constr. Build. Mater..

[10] Arora A., Sant G., Neithalath N. (2016). Ternary blends containing slag and interground/blended limestone: Hydration, strength, and pore structure. Constr. Build. Mater..

[11] Menéndez G., Bonavetti V., Irassar E.F. (2003). Strength development of ternary blended cement with limestone filler and blast-furnace slag. Cem. Concr. Compos..

[12] Stempkowska A., Gawenda T., Chajec A., Sadowski Ł. (2022). Effect of Granite Powder Grain Size and Grinding Time of the Properties of Cementitious Composites. Materials.

[13] Shamsabadi E.A., Ghalehnovi M., De Brito J., Khodabakhshian A. (2018). Performance of Concrete with Waste Granite Powder: The Effect of Superplasticizers. Appl. Sci..

[14] Ramezanianpour A.M., Hooton R.D. (2014). A study on hydration, compressive strength, and porosity of Portland-limestone cement mixes containing SCMs. Cem. Concr. Compos..

[15] Nehdi M., Mindess S. (1996). Opitimization of high strength limestonefiller cement mortars. Cem. Concr. Res..

[16] Ma J., Yu Z., Shi H., Zhang Y., Shen X. (2021). Long-term hydration behavior and pore structure development of cement–limestone binary system. J. Therm. Anal. Calorim..

[17] Matschei T., Lothenbach B., Glasser F. (2007). The role of calcium carbonate in cement hydration. Cem. Concr. Res..

[18] Madan C.S., Munuswamy S., Joanna P.S., Gurupatham B.G.A., Roy K. (2022). Comparison of the Flexural Behavior of High-Volume Fly AshBased Concrete Slab Reinforced with GFRP Bars and Steel Bars. J. Compos. Sci..

[19] Hay R., Peng B., Celik K. (2023). Filler effects of CaCO_3_ polymorphs derived from limestone and seashell on hydration and carbonation of reactive magnesium oxide (MgO) cement (RMC). Cem. Concr. Res..

[20] Rode S., Oyabu N., Kobayashi K., Yamada H., Kühnle A. (2009). True Atomic-Resolution Imaging of (10$\bar{1}$4) Calcite in Aqueous Solution by Frequency Modulation Atomic Force Microscopy. Langmuir.

[21] Araki Y., Tsukamoto K., Oyabu N., Kobayashi K., Yamada H. (2012). Atomic-Resolution Imaging of Aragonite(001)Surface in Waterby Frequency Modulation Atomic Force Microscopy. Jpn. J. Appl. Phys..

[22] Addadi L., Weiner S. (1992). Control and Design Principles in Biological Mineralization. Angew. Chem. Int. Ed..

[23] Camiletti J., Soliman A.M., Nehdi M.L. (2012). Effects of nano- and micro-limestone addition on early-age properties of ultra-high-performance concrete. Mater. Struct..

[24] Fu Q., Zhang Z., Zhao X., Xu W., Niu D. (2022). Effect of nano calcium carbonate on hydration characteristics and microstructure of cement-based materials: A review. J. Build. Eng..

[25] Wang D., Shi C., Farzadnia N., Shi Z., Jia H., Ou Z. (2018). A review on use of limestone powder in cement-based materials: Mechanism, hydration and microstructures. Constr. Build. Mater..

[26] Li L., Mingli C., Hong Y. (2019). Comparative roles between aragonite and calcite calcium carbonate whiskers in the hydration and strength of cement paste. Cem. Concr. Compos..

[27] Gong P., Zhang C., Wu Z., Zhang G., Mei K., Gao Q., Cheng X. (2022). Study on the effect of CaCO_3_ whiskers on carbonized self-healing cracks of cement paste: Application in CCUS cementing. Constr. Build. Mater..

[28] Ouyang X., Koleva D.A., Ye G., Van Breugel K. (2017). Understanding the adhesion mechanisms between C S H and fillers. Cem. Concr. Res..

[29] Menadi B., Kenai S., Khatib J., Aït-Mokhtar A. (2009). Strength and durability of concrete incorporating crushed limestone sand. Constr. Build. Mater..

[30] Ahmed A.H., Nune S., Liebscher M., Köberle T., Willomitzer A., Noack I., Butler M., Mechtcherine V. (2023). Exploring the role of dilutive effects on microstructural development and hydration kinetics of limestone calcined clay cement (LC3) made of low-grade raw materials. J. Clean. Prod..

[31] Cao M., Wei J. (2011). Microstructure and mechanical properties of CaCO_3_ whisker-reinforced cement. J. Wuhan Univ. Technol. Sci. Ed..

[32] Briki Y., Zajac M., Haha M.B., Scrivener K. (2021). Impact of limestone fineness on cement hydration at early age. Cem. Concr. Res..

[33] Juenger M.C.G., Siddique R. (2015). Recent advances in understanding the role of supplementary cementitious materials in concrete. Cem. Concr. Res..

[34] Alhozaimy A.M. (2009). Effect of absorption of limestone aggregates on strength and slump loss of concrete. Cem. Concr. Compos..

[35] He Z., Cai R., Chen E., Tang S. (2019). The investigation of early hydration and pore structure for limestone powder wastes blended cement pastes. Constr. Build. Mater..

[36] Sotiriadis K., Mazur A., Tolstoy P., Frankeová D. (2023). Chloride effect on sulfate attack in hydrated Portland-limestone cement assessed by 29Si NMR spectroscopy and thermal analysis. Mater. Today Proc..

[37] Cao M., Zhang C., Lv H., Xu L. (2014). Characterization of mechanical behavior and mechanism of calcium carbonate whisker-reinforced cement mortar. Constr. Build. Mater..

[38] Khan M., Cao M., Ali M. (2018). Effect of basalt fibers on mechanical properties of calcium carbonate whisker-steel fiber reinforced concrete. Constr. Build. Mater..

[39] Cao M., Ming X., He K., Li L., Shen S. (2019). Effect of Macro-, Micro- and Nano-Calcium Carbonate on Properties of Cementitious Composites—A Review. Materials.

[40] Karkhaneh S., Tarighat A., Jahromi S.G. (2023). Kinetics behavior of delayed ettringite in limestone calcined clay cement (LC3) by thermodynamic approach and consideration of the time factor. Constr. Build. Mater..

[41] Berodier E., Scrivener K. (2014). Understanding the Filler Effect on the Nucleation and Growth of C-S-H. J. Am. Ceram. Soc..

[42] Zhang Z., Chen W., Han F., Yan P. (2020). A new hydration kinetics model of composite cementitious materials, Part 2: Physical effect of SCMs. J. Am. Ceram. Soc..

[43] Pourchet S., Pochard I., Brunel F., Perrey D. (2013). Chemistry of the calcite/water interface: Influence of sulfate ions and consequences in terms of cohesion forces. Cem. Concr. Res..

[44] Scrivener K.L., Juilland P., Monteiro P.J. Advances in understanding hydration of Portland cement. Proceedings of the 14th International Congress on the Chemistry of Cement (ICCC 2015).

[45] Khan R.I., Ashraf W. (2019). Effects of ground wollastonite on cement hydration kinetics and strength development. Constr. Build. Mater..

[46] Myers R.J., Geng G., Li J., Rodríguez E.D., Ha J., Kidkhunthod P., Sposito G., Lammers L.N., Kirchheim A.P., Monteiro P.J.M. (2016). Role of Adsorption Phenomena in Cubic Tricalcium Aluminate Dissolution. Langmuir.

[47] Briki Y., Avet F., Zajac M., Bowen P., Ben Haha M., Scrivener K. (2021). Understanding of the factors slowing down metakaolin reaction in limestone calcined clay cement (LC3) at late ages. Cem. Concr. Res..

[48] Termkhajornkit P., Barbarulo R. (2012). Modeling the coupled effects of temperature and fineness of Portland cement on the hydration kinetics in cement paste. Cem. Concr. Res..

[49] Bellmann F., Scherer G.W. (2018). Analysis of C-S-H growth rates in supersaturated conditions. Cem. Concr. Res..

[50] Jönsson B., Nonat A., Labbez C., Cabane B., Wennerström H. (2005). Controlling the Cohesion of Cement Paste. Langmuir.

[51] Stark J., Möser B., Bellmann F. (2007). Nucleation and growth of C-S-H phases on mineral admixtures. Adv. Constr. Mater..

[52] Zajac M., Skocek J., Lothenbach B., Mohsen B.H. (2020). Late hydration kinetics: Indications from thermodynamic analysis of pore solution data. Cem. Concr. Res..

[53] Andalibi M.R., Kumar A., Srinivasan B., Bowen P., Scrivener K., Ludwig C., Testino A. (2018). On the mesoscale mechanism of synthetic calcium–silicate–hydrate precipitation: A population balance modeling approach. J. Mater. Chem. A.

[54] Garrault-Gauffinet S., Nonat A. (1999). Experimental investigation of calcium silicate hydrate (C-S-H) nucleation. J. Cryst. Growth.

[55] Yeşilmen S., Al-Najjar Y., Balav M.H., Şahmaran M., Yıldırım G., Lachemi M. (2015). Nano-modification to improve the ductility of cementitious composites. Cem. Concr. Res..

[56] Friebert M. (2005). Der Einfluss von Betonzusatzstoffen auf die Hydratation und Dauer-haftigkeit selbstverdichtender Betone. Ph.D. Thesis.

[57] Bentz D.P., Sato T., de la Varga I., Weiss W.J. (2012). Fine limestone additions to regulate setting in high volume fly ash mixtures. Cem. Concr. Compos..

[58] De Weerdt K., Haha M.B., Le Saout G., Kjellsen K.O., Justnes H., Lothenbach B. (2011). Hydration mechanisms of ternary Portland cements containing limestone powder and fly ash. Cem. Concr. Res..

[59] Kakali G., Tsivilis S., Aggeli E., Bati M. (2000). Hydration products of C3A, C3S and Portland cement in the presence of CaCO_3_. Cem. Concr. Res..

[60] Darweesh H.H.M. (2004). Limestone as an accelerator and filler in limestone-substituted alumina cement. Ceram. Int..

[61] Sato T., Beaudoin J.J. (2011). Effect of nano-CaCO_3_ on hydration of cement containing supplementary cementitious materials. Adv. Cem. Res..

[62] Mikanovic N., Khayat K., Pagé M., Jolicoeur C. (2006). Aqueous CaCO_3_ dispersions as reference systems for early-age cementitious materials. Colloids Surf. A Physicochem. Eng. Asp..

[63] Drissi S., Shi C., Li N., Liu Y., Liu J., He P. (2021). Relationship between the composition and hydration-microstructure-mechanical properties of cement-metakaolin-limestone ternary system. Constr. Build. Mater..

[64] Aqel M., Panesar D.K. (2016). Hydration kinetics and compressive strength of steam-cured cement pastes and mortars containing limestone filler. Constr. Build. Mater..

[65] Hu J. (2021). The Hydration Mechanism and the Improvement of Early Properties of Ternary Cement Blends with Limestone Powder and Metakaolin. Master’s Thesis.

[66] Zhou S.-C. (2019). Study on Evolutionary Regularity and Micro Mechanisms of Macro Properties of Composite Limestone Powder-Fly Ash-Slag Concrete. Ph.D. Thesis.

[67] Saulat H., Cao M., Khan M., Khan M.M., Rehman A. (2020). Preparation and applications of calcium carbonate whisker with a special focus on construction materials. Constr. Build. Mater..

[68] Yoshioka S., Kitano Y. (1985). Transformation of aragonite to calcite through heating. Geochem. J..

[69] Bentz D.P., Ardani A., Barrett T., Jones S.Z., Lootens D., Peltz M.A., Sato T., Stutzman P.E., Tanesi J., Weiss W.J. (2015). Multi-scale investigation of the performance of limestone in concrete. Constr. Build. Mater..

[70] Luan C., Zhou Y., Liu Y., Ren Z., Wang J., Yuan L., Du S., Zhou Z., Huang Y. (2022). Effects of nano-SiO_2_, nano-CaCO_3_ and nano-TiO_2_ on properties and microstructure of the high content calcium silicate phase cement (HCSC). Constr. Build. Mater..

[71] Goodbrake C.J., Young J.F., Berger R.L. (1979). Reaction of Beta-Dicalcium Silicate and Tricalcium Silicate with Carbon Dioxide and Water Vapor. J. Am. Ceram. Soc..

[72] Monkman S., Lee B.E.J., Grandfield K., MacDonald M., Raki L. (2020). The impacts of in-situ carbonate seeding on the early hydration of tricalcium silicate. Cem. Concr. Res..

[73] Monkman S., Sargam Y., Raki L. (2022). Comparing the effects of in-situ nano-calcite development and ex-situ nano-calcite addition on cement hydration. Constr. Build. Mater..

[74] Sato T., Diallo F. (2010). Seeding Effect of Nano-CaCO_3_ on the Hydration of Tricalcium Silicate. Transp. Res. Rec. J. Transp. Res. Board.

[75] Ding Y., Liu J.-P., Bai Y.-L. (2020). Linkage of multi-scale performances of nano-CaCO_3_ modified ultra-high performance engineered cementitious composites (UHP-ECC). Constr. Build. Mater..

[76] Xu Z., Zhou Z., Du P., Cheng X. (2017). Effects of nano-limestone on hydration properties of tricalcium silicate. J. Therm. Anal. Calorim..

[77] Douba A., Hou P., Kawashima S. (2023). Hydration and mechanical properties of high content nano-coated cements with nano-silica, clay and calcium carbonate. Cem. Concr. Res..

[78] Nguyen V.T., Lee S.Y., Kim D.J. (2023). Simulation of the effect of nano-CaCO_3_ agglomeration on the hydration process and microstructural evolution of cement paste. Case Stud. Constr. Mater..

[79] Shaikh F.U., Supit S.W. (2014). Mechanical and durability properties of high volume fly ash (HVFA) concrete containing calcium carbonate (CaCO_3_) nanoparticles. Constr. Build. Mater..

[80] Kawashima S., Seo J.W.T., Corr D., Hersam M.C., Shah S.P. (2013). Dispersion of CaCO_3_ nanoparticles by sonication and surfactant treatment for application in fly ash–cement systems. Mater. Struct..

[81] Wu Z., Shi C., Khayat K., Wan S. (2016). Effects of different nanomaterials on hardening and performance of ultra-high strength concrete (UHSC). Cem. Concr. Compos..

[82] Ming X., Cao M., Lv X., Yin H., Li L., Liu Z. (2020). Effects of high temperature and post-fire-curing on compressive strength and microstructure of calcium carbonate whisker-fly ash-cement system. Constr. Build. Mater..

[83] Berodier E.M.J. (2019). Impact of the Supplementary Cementitious Materials on the Kinetics and Microstructural Development of Cement Hydration. Ph.D. Thesis.

[84] Zajac M., Rossberg A., Le Saout G., Lothenbach B. (2014). Influence of limestone and anhydrite on the hydration of Portland cements. Cem. Concr. Compos..

[85] Zajac M., Durdzinski P., Stabler C., Skocek J., Nied D., Haha M.B. (2018). Influence of calcium and magnesium carbonates on hydration kinetics, hydrate assemblage and microstructural development of metakaolin containing composite cements. Cem. Concr. Res..

[86] Antoni M., Rossen J., Martirena F., Scrivener K. (2012). Cement substitution by a combination of metakaolin and limestone. Cem. Concr. Res..

[87] Dhandapani Y., Santhanam M., Kaladharan G., Ramanathan S. (2021). Towards ternary binders involving limestone additions—A review. Cem. Concr. Res..

[88] Tang J., Wei S., Li W., Ma S., Ji P., Shen X. (2019). Synergistic effect of metakaolin and limestone on the hydration properties of Portland cement. Constr. Build. Mater..

[89] Li L., Cao M., Xie C., Yin H. (2018). Effects of CaCO_3_ whisker, hybrid fiber content and size on uniaxial compressive behavior of cementitious composites. Struct. Concr..

[90] Cao M., Khan M., Ahmed S. (2020). Effectiveness of Calcium Carbonate Whisker in Cementitious Composites. Period. Polytech. Civ. Eng..

[91] Cao M., Xie C., Li L., Khan M. (2019). Effect of different PVA and steel fiber length and content on mechanical properties of CaCO_3_ whisker reinforced cementitious composites. Mater. Constr..

[92] Cao M., Li L., Khan M. (2018). Effect of hybrid fibers, calcium carbonate whisker and coarse sand on mechanical properties of cement-based composites. Mater. Constr..

[93] Khan M., Cao M., Ai H., Hussain A. (2022). Basalt Fibers in Modified Whisker Reinforced Cementitious Composites. Period. Polytech. Civ. Eng..

[94] Cao M., Khan M. (2020). Effectiveness of multiscale hybrid fiber reinforced cementitious composites under single degree of freedom hydraulic shaking table. Struct. Concr..

[95] Khan M., Cao M., Xie C., Ali M. (2022). Effectiveness of hybrid steel-basalt fiber reinforced concrete under compression. Case Stud. Constr. Mater..

[96] Li W., Huang Z., Cao F., Sun Z., Shah S.P. (2015). Effects of nano-silica and nano-limestone on flowability and mechanical properties of ultra-high-performance concrete matrix. Constr. Build. Mater..

[97] Papatzani S., Paine K., Calabria-Holley J. (2015). A comprehensive review of the models on the nanostructure of calcium silicate hydrates. Constr. Build. Mater..

[98] Jinchang P., Ronggui L. (2016). Improvement of performance of ultra-high performance concrete based composite material added with nano materials. Frat. Ed Integrità Strutt..

[99] Svenum I.-H., Ringdalen I.G., Bleken F.L., Friis J., Höche D., Swang O. (2020). Structure, hydration, and chloride ingress in C-S-H: Insight from DFT calculations. Cem. Concr. Res..

[100] Wu Z., Khayat K.H., Shi C., Tutikian B.F., Chen Q. (2021). Mechanisms underlying the strength enhancement of UHPC modified with nano-SiO_2_ and nano-CaCO_3_. Cem. Concr. Compos..

[101] Assaedi H., Alomayri T., Kaze C.R., Jindal B.B., Subaer S., Shaikh F., Alraddadi S. (2020). Characterization and properties of geopolymer nanocomposites with different contents of nano-CaCO_3_. Constr. Build. Mater..

[102] Irassar E.F. (2009). Sulfate attack on cementitious materials containing limestone filler—A review. Cem. Concr. Res..

[103] Ramezanianpour A.M., Hooton R.D. (2013). Thaumasite sulfate attack in Portland and Portland-limestone cement mortars exposed to sulfate solution. Constr. Build. Mater..

[104] Yang Z., Zhang W., Zhu H., Chen Y., Xu L., Wang P., Lai Y. (2023). Thaumasite form of sulfate attack in ettringite rich-ternary systems: Effects of limestone filler, etching solutions and exposure temperature. Dev. Built Environ..

[105] Gunjal S., Turkane S.D., Patankar S., Kondraivendhan B. (2023). Effect of magnesium sulphate and sulphuric acid attack on limestone calcined clay cement concrete. Mater. Today Proc..

[106] Sun J., Chen Z. (2018). Influences of limestone powder on the resistance of concretes to the chloride ion penetration and sulfate attack. Powder Technol..

[107] Rashad A.M., Ezzat M., ElNagar A.M., El-Nashar M. (2023). Valorization of limestone powder as an additive for fly ash geopolymer cement under the effect of the simulated tidal zone and seawater attack. Constr. Build. Mater..

[108] Nadelman E., Kurtis K. (2019). Durability of Portland-limestone cement-based materials to physical salt attack. Cem. Concr. Res..

[109] Kanagaraj B., Nammalvar A., Andrushia A.D., Gurupatham B.G.A., Roy K. (2023). Infuence of Nano Composites on the Impact Resistance of Concrete at Elevated Temperatures. Fire.

[110] Cao M., Yuan X., Ming X., Xie C. (2022). Effect of High Temperature on Compressive Strength and Microstructure of Cement Paste Modified by Micro- and Nano-calcium Carbonate Particles. Fire Technol..

[111] Kunther W., Dai Z., Skibsted J. (2016). Thermodynamic modeling of hydrated white Portland cement–metakaolin–limestone blends utilizing hydration kinetics from 29Si MAS NMR spectroscopy. Cem. Concr. Res..

[112] Liu X., Luo Q., Xie H., Li S., Zhang J., Xia C., Ding Y., Chen Y., Gao R., Wei Z. (2023). Effect of calcium alumina silicate hydrate nano-seeds on the hydration of low clinker cement. J. Build. Eng..

[113] Matschei T., Lothenbach B., Glasser F.P. (2007). Thermodynamic properties of Portland cement hydrates in the system CaO–Al_2_O_3_–SiO_2_–CaSO_4_–CaCO_3_–H_2_O. Cem. Concr. Res..

[114] Naber C., Bellmann F., Sowoidnich T., Goetz-Neunhoeffer F., Neubauer J. (2019). Alite dissolution and C-S-H precipitation rates during hydration. Cem. Concr. Res..

[115] Schöler A., Lothenbach B., Winnefeld F., Ben Haha M., Zajac M., Ludwig H.-M. (2017). Early hydration of SCM-blended Portland cements: A pore solution and isothermal calorimetry study. Cem. Concr. Res..

[116] Lothenbach B., Kulik D.A., Matschei T., Balonis M., Baquerizo L., Dilnesa B., Miron G.D., Myers R.J. (2019). Cemdata18: A chemical thermodynamic database for hydrated Portland cements and alkali-activated materials. Cem. Concr. Res..

[117] Lothenbach B., Winnefeld F. (2006). Thermodynamic modelling of the hydration of Portland cement. Cem. Concr. Res..

[118] Parkhurst D.L., Appelo C.A.J. (2013). Description of Input and Examples for PHREEQC Version 3—A Computer Program for Speciation, Batch-Reaction, One-Dimensional Transport, and Inverse Geochemical Calculations. US Geol. Surv. Tech. Methods.

[119] Damidot D., Lothenbach B., Herfort D., Glasser F. (2011). Thermodynamics and cement science. Cem. Concr. Res..

[120] Elizalde M., Aparicio J. (1995). Current theories in the calculation of activity coefficients—II. Specific interaction theories applied to some equilibria studies in solution chemistry. Talanta.

[121] Lothenbach B., Le Saout G., Gallucci E., Scrivener K. (2008). Influence of limestone on the hydration of Portland cements. Cem. Concr. Res..

[122] Wang Y., Shui Z., Wang L., Gao X., Huang Y., Song Q., Liu K. (2020). Alumina-rich pozzolan modification on Portland-limestone cement concrete: Hydration kinetics, formation of hydrates and long-term performance evolution. Constr. Build. Mater..

[123] Kunther W., Dai Z., Skibsted J. Thermodynamic modeling of Portland cement-metakaolin-limestone blends. Proceedings of the 14th International Congress on the Chemistry of Cement (ICCC 2015).

[124] Her S., Im S., Liu J., Suh H., Kim G., Sim S., Wi K., Park D., Bae S. (2024). Exploring the potential of pulverized oyster shell as a limestone substitute in limestone calcined clay cement (LC3) and its implications for performance. Constr. Build. Mater..

[125] Fakhri R.S., Dawood E.T. (2023). Limestone powder, calcined clay and slag as quaternary blended cement used for green concrete production. J. Build. Eng..

[126] Mañosa J., Calderón A., Salgado-Pizarro R., Maldonado-Alameda A., Chimenos J.M. (2024). Research evolution of limestone calcined clay cement (LC3), a promising low-carbon binder—A comprehensive overview. Heliyon.

[127] Frías M., Guerrero A., Monasterio M., Insignares Á., de Rojas M.I.S. (2024). Viability of using limestone concrete waste from CDW to produce ternary cements type LC3. Constr. Build. Mater..

[128] Hanein T., Thienel K.-C., Zunino F., Marsh A.T.M., Maier M., Wang B., Canut M., Juenger M.C.G., Ben Haha M., Avet F. (2021). Clay calcination technology: State-of-the-art review by the RILEM TC 282-CCL. Mater. Struct..

[129] Cai J.M., Pan J.L. Using Calcium Carbonate Whisker in Engineered Cementitious Composites. Proceedings of the 9th International Conference on Fracture Mechanics of Concrete and Concrete Structures.

[130] Xie C., Cao M., Si W., Khan M. (2020). Experimental evaluation on fiber distribution characteristics and mechanical properties of calcium carbonate whisker modified hybrid fibers reinforced cementitious composites. Constr. Build. Mater..

[131] Kim G., Cho S., Moon J., Suh H., Her S., Sim S., Bae S. (2024). Investigation of the hydrate formation and mechanical performance of limestone calcined clay cement paste incorporating nano-CaCO_3_ and nano-SiO_2_ as partial limestone substitutes. Constr. Build. Mater..

[132] Meng S., Ouyang X., Fu J., Ma Y., Ye G. (2021). New insights into the role of MWCNT in cement hydration. Mater. Struct..

[133] Zhang R., Scott A.N., Panesar D.K. (2024). Carbonation and CO_2_ reabsorption of cement-based materials: Influence of limestone filler and ground-granulated blast-furnace slag. Constr. Build. Mater..

[134] Kim G., Kurtis K.E. (2022). Early-stage assessment of drying shrinkage in Portland limestone cement concrete using nonlinear ultrasound. Constr. Build. Mater..

[135] Barbhuiya S., Nepal J., Das B.B. (2023). Properties, compatibility, environmental benefits and future directions of limestone calcined clay cement (LC3) concrete: A review. J. Build. Eng..

